# Using the Stressor-System-Resilience Resources-Outcomes (SSRO) scaffold to clarify the core components of resilience research in medicine

**DOI:** 10.1038/s43856-026-01864-4

**Published:** 2026-08-15

**Authors:** Deepak K. Ravi, Kaisa Koivunen, Kirsten DJ Bos, René JF Melis

**Affiliations:** 1 Institute for Biomechanics, ETH Zurich, Zurich, Switzerland; 2 Faculty of Sport and Health Sciences, and Gerontology Research Center, University of Jyväskylä, Jyväskylä, Finland; 3 Department of Geriatrics, Radboud university medical center, Nijmegen, The Netherlands; 4 Clinical Research Centre, Rijnstate Hospital, Arnhem, The Netherlands

**Keywords:** Epidemiology, Prognosis

## Abstract

Modern medicine increasingly extends beyond treating disease to supporting individuals through recovery, adaptation, and long-term functioning, all central to the concept of resilience. Despite growing interest, resilience remains conceptually difficult to structure and compare across disciplines, limiting its value for guiding research and clinical care. In this Perspective, we identify recurring features across many resilience traditions and propose a practical organizing scaffold for studying resilience in medicine. The scaffold specifies four elements that any resilience study should make explicit: the stressor, the system, resilience-relevant resources and processes, and outcomes. Using worked examples, we show how the scaffold clarifies resilience questions and improves comparability across studies. We also provide a step-by-step roadmap to help researchers new to the field design resilience studies, from framing a question through to interpreting results.

## Abstract (web summary)

Ravi et al. propose the SSRO scaffold, identifying four elements, stressor, system, resources, and outcomes, needed to study resilience consistently across medicine. Worked examples from ageing and neurology show how the scaffold clarifies resilience questions, and a step-by-step roadmap guides study design for researchers new to the field.

## Introduction

For much of modern medical history, health has been defined narrowly, first as the absence of disease and later as optimally maintained physical, mental and social well-being^1^. However, in an aging society where chronic conditions and multimorbidity are increasingly common, this definition no longer serves us. Increasingly, health is being reconceptualized as the capacity to adapt to and recover from the physical, cognitive, and social disruptions that emerge across the life span^2^. Although not explicitly labeled as such, this perspective frames health as the ability to resist, recover from, or adapt to challenges, which is now commonly referred to as ‘resilience’^3^. This evolving view carries important implications not only for how physical health is assessed, but also for how it is managed.

Traditionally, medicine has prioritized the mitigation or elimination of disease and risk as the primary path to restoring and maintaining health. While essential, this focus often overlooks the capacity of the system under consideration (most often the individual) to resist, recover, or adapt to health risks and stressors. Incorporating this systems-level capacity for resilience, much of which has been shaped through evolutionary processes, into our definition of health expands the possibilities for management. It enables strategies that go beyond the prevention and treatment of disease to include support for the system’s adaptive and recovery processes in response to health stressors.

These shifts have positioned resilience as an increasingly prominent concept in both fundamental and clinical research, yet resilience research remains difficult to structure and compare across medical fields, health-related disciplines, and adjacent research traditions. Such inconsistencies, and the controversies they generate, may hinder the development of knowledge and the implementation of resilience assessment and management in medicine.

In this Perspective, we explore why these inconsistencies persist and consider how greater conceptual clarity may help move the field forward in medicine. To support this aim, we clarify the minimal elements that need to be specified when resilience concepts are applied in medical research and care, and offer a simple organizing scaffold to help structure, compare, and communicate existing resilience approaches in medicine more clearly. This perspective was developed through iterative interdisciplinary discussions among the co-authors, informed by their complementary expertise in geriatrics, rehabilitation, aging, and resilience research. It was further informed by synthesis of resilience research from fields within and adjacent to medicine, including psychology, ecology, and engineering, with a particular focus on insights applicable to somatic health.

### A proliferating field of diverging definitions and frameworks

Recently, efforts to define and operationalize resilience have increasingly diverged across fields. Different researchers, starting from different goals and conceptual traditions, have introduced a wide array of definitions, metrics, and frameworks for studying resilience^4–6^. Developmental and psychological perspectives have emphasized resilience as positive adaptation in the context of adversity, often highlighting dynamic processes that unfold over time^7–10^. Social-ecological perspectives have further stressed that resilience depends not only on the individual but also on access to social, environmental, and cultural resources^11,12^. Physiological traditions, including allostasis and allostatic load, have framed adaptation in terms of dynamic regulation and the cumulative costs of repeated stress responses^13,14^. Ecological perspectives, in turn, have emphasized resilience as the amount of disturbance a complex system can tolerate before shifting to another stable state. This tradition also makes clear that resilience is not inherently positive, since systems may remain resilient in both desirable and undesirable states^15,16^. While each perspective offers valid insights, the resulting diversity has created a fragmented field in which definitions risk competing rather than converging.

This challenge has been recognized previously^15^ and is not unique to resilience; similar patterns of conceptual proliferation have been observed in the study of frailty and stress^14,17^. In the case of frailty, for example, the emergence of physical, cognitive, and social variants has complicated rather than clarified our understanding, making it difficult to compare studies, design interventions, or communicate meaningfully across disciplines^18^.

### The risk of fragmentation in applying the resilience concept to medicine

For the application of resilience in medicine, it is important to prevent it from following the same fragmented trajectory observed in other constructs. The introduction of labels such as “psychological resilience” and “physical resilience” may be strategically useful for sensitizing the medical field to the value of resilience thinking^19,20^. Yet, introducing these labels may create urgency within the research community to define or operationalize them for research and clinical purposes as distinct constructs requiring scientific definition and measurement. Tools such as the Physical Resilience Scale^21^ and the Physical Resilience Instrument for Older Adults (PRIFOR)^22^ illustrate this well. Both operationalize resilience as the capacity to cope with physical health stressors. While useful, such operationalizations capture only part of what resilience may encompass in medicine.

We do not argue against rigorous conceptualization of resilience within specific disciplines; such efforts are crucial for developing valid research approaches. However, because multiple dimensions of resilience must be carefully defined and measured, oversimplifying these into singular, neatly operationalized constructs, such as “physical” or “psychological” resilience, can be counterproductive. This is particularly evident in medicine, where resilience operates across hierarchical levels of biological organization, with changes emerging first at the cellular and physiological level before manifesting at the functional level^23^. Operationalizing resilience at only one of these levels therefore risks misrepresenting the underlying process.

### The case for conceptual integration

To illustrate this, consider what may initially appear as a clear separation between psychological and physical resilience. Attempting to scientifically distinguish between the two quickly reveals a fundamental ambiguity: are we classifying resilience based on the type of stressor, the system involved, the resources that confer resilience, or the nature of the outcome? In practice, it can be either. A psychological threat, such as concern or fear of falling, can increase the risk of future falls and related physical harm^24^; likewise, a physical insult may have cognitive or emotional consequences^25^. Health challenges are inherently biopsychosocial in nature and rarely conform to disciplinary boundaries^26,27^. Operationalizations of resilience for medicine need to accommodate this.

Nevertheless, the field continues to produce narrowly defined operationalizations, which risk undermining the very utility of resilience as a unifying concept. Rather than pursuing ever finer distinctions, we advocate for a broader and more integrated way of structuring resilience research that can accommodate the multiple interacting dimensions of human adaptation.

### Measuring resilience in practice

One of the most persistent challenges in resilience research is that the term “resilience” is used to refer to many different things. It is variously described as a trait, a capacity, a process, or an outcome, often even within the same body of literature. Depending on the context, researchers have argued that resilience should be defined exclusively as one of these aspects, despite the fact that all are relevant to studying and understanding resilience. This definitional ambiguity has not only hindered the development of consistent metrics but has also made it difficult to interpret findings or translate them into clinical practice. Clarifying what it means to study resilience in the context of human health requires more than semantic alignment; it calls for a coherent yet flexible scaffold, one that specifies the conditions under which resilience can be observed, measured, and meaningfully compared, and that identifies the essential elements involved. To generate findings that can be compared and translated across settings, the elements that constitute resilience need to be defined unequivocally.

Different traditions across resilience research have emerged from diverse disciplines, each with its own concepts, terminology, and points of emphasis. Yet the challenge is often not that these traditions describe entirely different phenomena, but that they use different language to describe overlapping conceptual components. Despite this variation, many resilience approaches converge around a small set of recurring elements (Supplementary Data 1; see Supplementary Methods 1 for literature screening informing this synthesis) that need to be made explicit for resilience to be studied clearly and consistently in medicine. Building on prior work across resilience research, including the trans-NIH Resilience Working Group framework^27^ and other lines of work, such as those on pain resilience^4^, we argue that resilience research in medicine benefits from a simple cross-cutting structure. Unlike many domain-specific frameworks summarized in Supplementary Data 1, which are pitched within a particular field and rich in that field’s own constructs, the scaffold proposed here is deliberately minimal: it specifies the elements that must be defined for resilience to be studied at all, regardless of discipline. This minimalism allows the scaffold to function as a common reference structure that the traditions summarized in Supplementary Data 1 can be mapped onto, making it possible to translate between traditions rather than requiring researchers to adopt one over another.

**Fig. 1:**
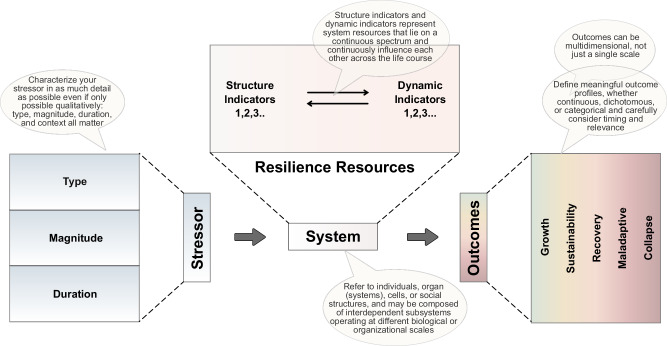
The SSRO scaffold outlines the key components for studying resilience in medicine. The scaffold (Stressor, System, Resilience-relevant resources and processes, Outcome) emphasizes the need to characterize the stressor (including its type, magnitude, duration, and context), identify the system and its relevant resources and processes (including structure indicators and dynamic indicators), and define meaningful outcomes of interest. Together, these four elements provide a simple yet flexible organizing structure for resilience research, ensuring clarity, comparability, and applicability across domains.

**Fig. 2:**
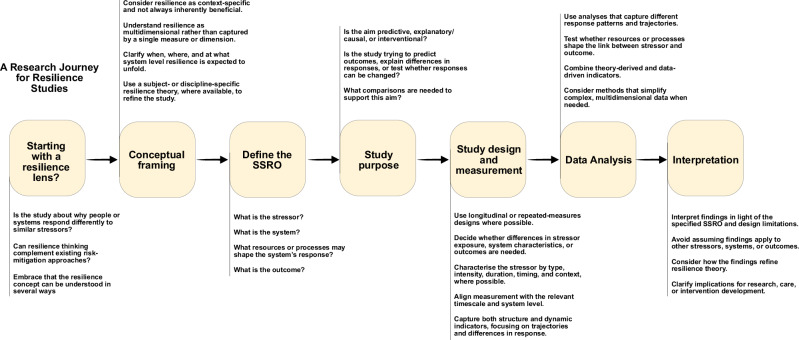
A research journey for resilience studies. This figure illustrates a structured, step-by-step roadmap guiding researchers through the key methodological decisions involved in conducting resilience research. At each stage, guiding questions and practical considerations are provided to help researchers navigate common challenges, including conceptualizing resilience as multidimensional and context-specific, aligning measurement with theory, and drawing meaningful, appropriately bounded conclusions. This roadmap is intended as an accessible entry point for scholars new to resilience research, supporting rigorous and theoretically grounded study design.

At a minimum, this structure should specify (1) the stressor or challenge, (2) the system under consideration (often the individual), (3) the resilience-relevant resources and processes within or around the system that are hypothesized to shape its response to the stressor, and (4) the outcomes of interest (Fig. 1). We refer to this organizing scaffold as the SSRO - Stressor, System, Resilience-relevant resources/processes, and Outcomes. These four elements form the essential building blocks of resilience research. Each may be physical, psychological, cognitive, or social in nature, and they often interact across domains. The SSRO scaffold is designed as a simple and memorable organizing scaffold and educational tool to promote clarity, comparability, and rigor in resilience research. To help researchers new to the field translate these elements into practice, we also provide a step-by-step research journey roadmap (Fig. 2).

### Stressors

Stressors refer to any form of stress, adversity, perturbation, or challenge that is hypothesized to affect the functioning or integrity of a system. They can be characterized by their type, magnitude, and duration, but their relevance and interpretation depend on the context in which they occur and the specific demands they place on the system. Multiple stressors may act simultaneously, and their effects are not necessarily negative—exercise, for example, can also be viewed as a stressor.

The terminology used to describe the stressor varies considerably across resilience traditions, reflecting differences in disciplinary context. In human resilience research, the terms adversity, challenge, and stressor are most common, while the ecology literature favors perturbation, and engineering uses shock or crisis. Some traditions also frame the stressor as a risk^28,29^. This terminological variation has substantive implications. Framing the stressor as a risk connects resilience research to risk management, where proactive preparedness aims to reduce the likelihood or impact of an adverse event before it occurs. Resilience management, in contrast, focuses on reactive preparedness - minimizing the impact of a stressor once it materializes. These two approaches are complementary rather than competing^30^. Additionally, the word stressor implies a temporal dimension, a discrete exposure with a beginning and an end, whereas the word risk carries no such implication. This distinction underscores that stressors require adequate characterization not only in terms of type and magnitude, but also duration. Finally, framing the stressor as a risk ties it to questions of causality. While the literature on causal evaluation of risk factors is well developed, causal study of resilience factors remains sparse, with most resilience research oriented toward prediction rather than causal inference. Yet for the purposes of intervention, understanding the causal role of resilience factors and mechanisms is essential.

### System

The system defines the entity whose response to a stressor is being examined. In clinical research, this often refers to the individual, yet resilience can also be investigated at the level of physiological subsystems (e.g., muscular, neural, cardiovascular) or within broader social or environmental contexts. Clearly defining the system establishes the scope of observation and ensures that resilience characteristics are interpreted at the appropriate biological or contextual scale.

There is general consensus in the resilience literature that resilience is not a fixed individual trait, but rather a process shaped by the ongoing interaction between the individual and their environment across space and time. Nevertheless, research practice does not always reflect this consensus, and resilience is sometimes studied through a set of cross-sectionally collected individual characteristics. When defining the system, it is therefore important to consider not only the individual and their internal resources, but also the broader ecological, social, and temporal context in which they are embedded. Omitting this context risks reducing resilience to a property of the person alone, which both misrepresents the underlying process and limits the scope for intervention.

### Resilience-relevant resources and processes

In principle, these refer to features of the system of interest that are hypothesized to shape how the system responds to a stressor. However, resources external to the system, such as social welfare support or community infrastructure, may also enhance its resilient response. Within the SSRO scaffold, such external factors can be considered contextual resources that interact with, rather than belong to, the system itself, depending on the exact delineation of the system of interest. We also propose distinguishing between resilience-relevant resources and processes along a spectrum from more structural or static indicators to more dynamic indicators.

Structure indicators refer to latent or directly observable capacities of the system that can be assessed in the absence of a stressor. These are indicators such as muscle mass, educational attainment, or social network size. In the context of resilience in geriatric medicine, these indicators may reflect well-established constructs such as frailty, intrinsic capacity, or the biological pillars of aging, depending on the specific application. Dynamic indicators, in contrast, become evident only when the system’s functioning is assessed under challenge or when a person is exposed to a stressor, for example, gait adaptation following a perturbation or emotional regulation under pressure. While structure indicators provide valuable insight into a person’s potential to respond, they may not be sufficient on their own to fully capture resilience, because some vulnerabilities may remain hidden until the system is challenged^31^. It is the quality of dynamic indicators, emerging from the system’s response to stressors, that most clearly reveals the essence of resilience. These indicators may be derived from standardized assessments (e.g., stress or fatigability tests) or time series-based features that reflect adaptation to naturally occurring stressors.

However, the distinction between structure indicators and dynamic indicators is intended as a pedagogical tool rather than a definitive classification. In real systems, strict dichotomy is rarely possible and aiming for a complete categorization of indicators as either structural or dynamic is neither realistic nor desirable. Instead, these factors can be positioned along a continuum, ranging from more structural to more dynamic (Fig. 1). Whether a given resource appears structural or dynamic depends also on the context and the timescale of observation. Moreover, different resources may be involved at different time points in relation to the stressor. This highlights the importance of incorporating time scale into the definition and operationalisation of a resilience study.

### Outcomes

Outcomes extend beyond a single endpoint and may be defined in terms of timing, relevance, and trajectories such as recovery, sustained function, maladaptation, or collapse. They can also encompass growth or transformation, where a system emerges strengthened or more adaptive following adversity. Importantly, resilience does not always imply a desirable state—systems can be highly resilient within maladaptive or unwanted configurations, maintaining stability despite dysfunction. Moreover, different outcome domains may diverge; for instance, a treatment might restore physical health while leaving lasting psychological distress. Recognizing such multidimensional outcomes ensures that resilience is not equated solely with recovery but understood as a spectrum of potential trajectories.

An important consideration in operationalizing outcomes concerns the valence attributed to resilience. In everyday language, and often in psychological and stress resilience research, resilience is understood as inherently positive, an outcome is only considered resilient if it involves growth or improvement beyond pre-adversity functioning. However, this positive valence is not a universal feature of resilience. In ecological conceptualizations, both desired and undesired stable states can be resilient, since resilience refers to the persistence of a system state, not its desirability^11,15^. This has meaningful clinical implications. There is no such thing as a universally resilient person; resilience is not a static, unitary property exclusive to those without deficits or pathology^7,32,33^. A frail person can exhibit resilience, and that process can be supported. Even responses that appear maladaptive or normatively deviant in one domain may serve to maintain integrity in another domain or in the system as a whole^34,35^. These considerations further underscore the importance of explicit and careful operationalization of outcomes in resilience research.

A related concept that merits attention is the distinction between psychological immunity and psychological elasticity, which connects to the broader phenomenon of steeling versus sensitization^36^. The same adversity may either strengthen the system’s response repertoire, steeling, or weaken it through sensitization. This means that exposure to a stressor does not have a fixed or predictable effect on future resilience; the direction depends on the nature of the stressor, the system, and the context. Recognizing this bidirectionality reinforces the importance of longitudinal outcome assessment and careful specification of the timeframe over which resilience is evaluated.

### Study design considerations for resilience research

While this scaffold can be applied for both explanatory (etiological) and predictive purposes, the appropriate study design depends on the primary goal. From an explanatory or causal perspective, resilience-relevant resources and processes can often be conceptualized as effect modifiers: factors that alter the relationship between a stressor and its outcome. Studying resilience causally therefore requires sufficient contrast in exposure to the stressor, or in stressor intensity, to determine whether a given resource or process modifies the impact of the stressor rather than merely predicting the outcome. Without such contrast, it becomes difficult to distinguish resilience mechanisms from general prognostic factors. Some resilience mechanisms, moreover, may only manifest in the presence of the stressor, much like an immune response that can only be observed once the system is challenged. In contrast, if the goal is predictive, for example to identify who is likely to recover or adapt following a stressor, an unexposed control group may not always be necessary, particularly when no meaningful change is expected in the absence of stress. In such cases, the emphasis shifts from causal identification to accurate prediction, validation, and clinical utility.

In practice, however, applying this distinction can be challenging, particularly in clinical or epidemiological settings where stressors are often sudden and baseline data on potential resilience resources may be unavailable. In such cases, researchers may need to make compromises in design or interpretation, using the scaffold as a flexible guide rather than a rigid standard, yet highlighting the possible limitations of their design. Studying resilience epidemiologically also requires careful attention to contrasts, not only in exposure and outcome, but also in system’s resources, since such contrasts are the basis for inference. Depending on the goal, traditional designs like cohort studies (sampled by exposure) or case-control studies (sampled by outcome) may be adapted to resilience research but should be aligned with whether the aim is explanatory or predictive.

Finally, the meaning of a measure depends not only on what is assessed, but also on how, when, and where it is assessed. The complexity increases further when we consider interactions across domains. Physical, psychological, cognitive, and social resources and processes do not operate in isolation; their relationships are nonlinear, temporally variable, and context dependent. As the number and complexity of these interactions grow, traditional methods in epidemiology and causal inference may struggle to capture meaningful system-level patterns. For example, identifying profiles of multiple interacting factors that shape resilience cannot be adequately addressed using conventional interaction terms or stratified models. In such cases, it may be more productive to shift the focus from isolated components toward the emergent behavior of the system as a whole. From this perspective, resilience is not simply the sum of its parts but a dynamic, emergent property of a system responding to challenges. Future work informed by complex systems theory may help generate new hypotheses for resilience research^37^, for example, whether changes in network connectivity or fluctuations within or between domains might indicate loss or recovery of resilience in a system.

The considerations outlined in this section are summarized in Fig. 2, which may serve as a practical guide when designing a resilience study.

### Illustrative examples

To illustrate how the SSRO scaffold can be applied in practice, we present three examples: two from ageing research, addressing resilience in the context of social isolation and recovery following acute illness, and a third from neurology, addressing resilience to freezing of gait in Parkinson’s disease.

#### Example 1 Resilience amid COVID-19-related social distancing

The study by Koivunen et al. (2022) examined factors that promote high quality of life (QoL) among community-dwelling older adults in Finland during COVID-19-related social distancing^38^. Resilience was operationalized using an “a priori” method^39^, whereby participants were categorized into groups based on researcher-defined thresholds for levels of adversity and outcome, to identify those presumed to demonstrate resilience.

**Fig. 3:**
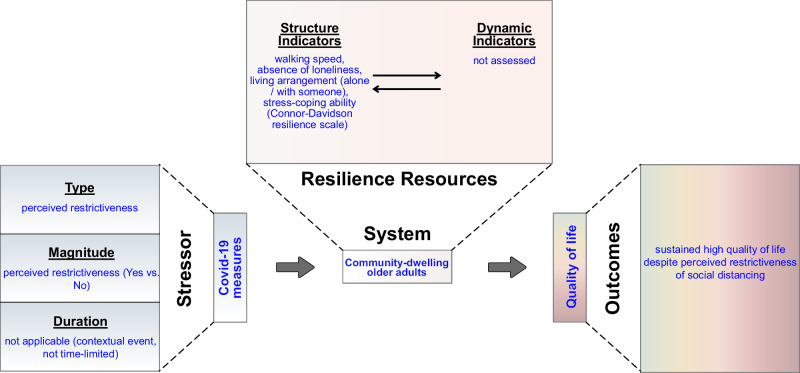
Mapping the study by Koivunen et al. (2022) onto the Stressor–System–Resilience Resources–Outcomes (SSRO) scaffold. This example illustrates how a study on quality of life among community-dwelling older adults during COVID-19-related social distancing can be characterized using the four SSRO elements, highlighting the distinction between structure and dynamic indicators of resilience resources and the absence of dynamic measurement in this study.

The stressor was the subjective severity of social distancing, measured by how much participants perceived the social distancing recommendations limited their desired activities (Fig. 3). Responses were dichotomized into “no perceived restrictiveness” and “perceived restrictiveness.” Combining perceived restrictiveness and QoL produced four categories, including a key subgroup (perceived restrictiveness + high QoL) indicating resilience. This design allowed testing of the buffering hypothesis^40^, which suggests that certain resources are particularly important for positive outcomes when individuals are exposed to stressors.

The selection of resources relied on a socioecological framework of resilience, which instead of viewing resilience solely as an individual trait, conceptualizes resilience as a process shaped by the individual and multiple levels of their surrounding environment. Accordingly, the study focused on structure indicators at the individual, social, and environmental levels, all measured two years prior to the onset of social distancing, which was possible because this was part of an already ongoing cohort study. Individual resources included stress-coping ability (assessed using a psychological resilience scale) and walking speed (a proxy for physiological reserve). Social connectedness was captured by absence of loneliness, and environmental support by cohabitation status, presumed to offer emotional and practical help.

#### Example 2 Predicting resilience after acute illness in older adults

**Fig. 4:**
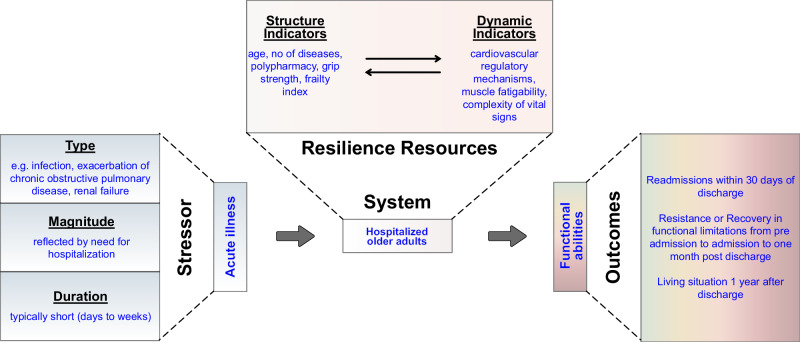
Mapping the “Predictors of Physical Resilience at the AMU” study onto the SSRO scaffold. This example illustrates how a predictive study of functional recovery following acute illness in hospitalized older adults can be characterized using the four SSRO elements, and notably demonstrates a study in which both structure and dynamic indicators of resilience resources were assessed, enabling a more comprehensive characterization of physiological adaptability.

In the “Predictors of Physical Resilience at the AMU” study, structure and dynamic indicators were examined to assess their ability to predict functional outcomes following acute illness in older adults admitted to the acute medical unit (AMU) at Radboudumc. The stressor was defined as acute illness, encompassing a broad range of conditions (e.g., infection, COPD exacerbation, acute renal failure) across multiple AMU specialties (Fig. 4). The magnitude of illness was indicated by the need for hospitalization and the Modified Early Warning Score at admission. The duration of the stressor was typically limited to a short period (days to weeks). The study followed a predictive design, aiming to identify indicators associated with recovery outcomes. All participants experienced an acute illness, and no experimental contrast or manipulation of the stressor was introduced.

A comprehensive multisystem assessment was conducted. Structure indicators were captured, including age, multimorbidity, polypharmacy, frailty (measured using the Clinical Frailty Scale, Frailty Index, and grip strength), and mental resilience as a trait (assessed via the Brief Resilience Scale). Dynamic indicators were derived to reflect physiological adaptability, including cardiovascular responses to a sit-to-stand challenge, muscle fatigability (grip work test), and vital sign time series characteristics (e.g., critical slowing down, loss of complexity)^41^.

Outcomes were centered on physical function, with resilience conceptualized as the ability to resist or recover from decline. Resistance was inferred from changes in daily functioning from two weeks prior to admission, while recovery was assessed at one- and three-months post-discharge. Sustained functioning was evaluated through 30-day readmission rates and living situation at one year. Indicators such as poor recovery, unplanned readmissions, institutionalization, or premature death were interpreted as signs of compromised resilience or system collapse.

#### Example 3 Resilience to freezing of gait in Parkinson’s disease

**Fig. 5:**
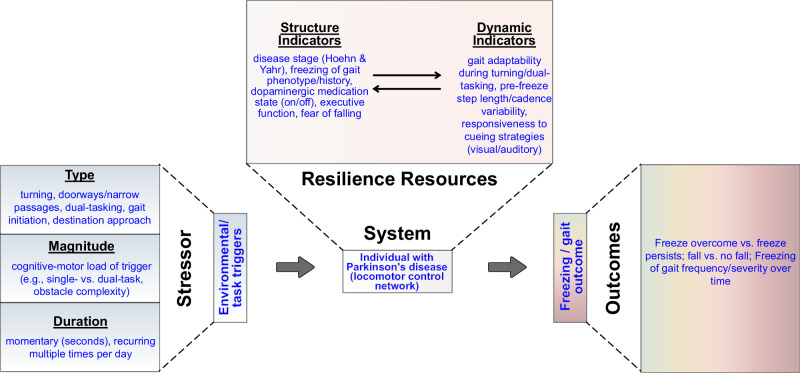
Mapping resilience to freezing of gait in Parkinson’s disease onto the SSRO scaffold. This example illustrates how a brief, recurring, task- and environment-triggered stressor can be characterized using the four SSRO elements, and demonstrates how both structure and dynamic indicators of resilience resources jointly shape whether a freezing episode is overcome or persists, extending the scaffold’s applicability beyond the longer-timescale stressors featured in Examples 1 and 2.

Freezing of gait (FOG) is a common and disabling symptom of Parkinson’s disease, characterized by a sudden, brief inability to generate effective stepping despite the intention to walk. Unlike the stressors in Examples 1 and 2, which are prolonged and largely externally imposed, FOG-triggering stressors are momentary and recur repeatedly within a single day: turning, walking through doorways, dual-tasking, and approaching a destination are all well-established provocateurs (Fig. 5)^42^.

The system under consideration is the individual’s locomotor control network, encompassing cortico-basal ganglia-brainstem circuits for gait initiation and task-switching, which in Parkinson’s disease operate under dopaminergic depletion and reduced automaticity, placing greater demand on attentional and executive resources during gait.

Structure indicators relevant to FOG resilience include disease stage, FOG phenotype and history, dopaminergic medication state, executive function, and fear of falling, all of which shape a person’s underlying vulnerability to freezing. Dynamic indicators become observable only when the system is challenged by a trigger: gait adaptability during turning or dual-tasking, pre-freeze changes in step length and cadence variability, and the effectiveness of compensatory cueing strategies, such as visual floor markers or auditory rhythmic cues, in restoring stepping. These dynamic indicators most directly capture resilience, since two people with similar disease severity can respond very differently to the same trigger: one recovers stepping within a stride using a cueing strategy, while another enters a prolonged freeze culminating in a near-fall or fall.

Outcomes can therefore be defined at the level of a single episode (resolution versus persistence of the freeze, and whether a fall results) or aggregated over time (FOG frequency and severity, and downstream effects on mobility-related quality of life). This example illustrates how the SSRO scaffold accommodates stressors that are brief and repeated, extending the scaffold’s applicability beyond the longer-timescale stressors featured in Examples 1 and 2.

## Conclusion

Resilience offers a promising lens for understanding how individuals navigate health challenges across the course of life. To translate this promise into practice, resilience research would benefit from simple, clear tools that support more consistent study design and reporting. We propose that resilience research can be organized around four core elements: a defined stressor, a specified system, the resilience-relevant resources and processes that may shape how the system responds, and the outcomes that follow. We refer to this organizing scaffold as SSRO. These components must be considered across physical, psychological, cognitive, and social domains, and in relation to the context in which adaptation occurs. Using the SSRO scaffold as a starting point, researchers can detail their study design according to the resilience tradition that best fits their use case. For those new to the field, we additionally offer a step-by-step research journey roadmap to guide key methodological decisions, from conceptual framing to interpretation.

In the future, advancing the field will ultimately require consensus among experts to establish unequivocal operational definitions for each of the four SSRO elements. Such agreement may not yet be necessary for conceptual progress but will likely be important for developing standardized measures, enabling data synthesis, and improving comparability across studies. Future work should also evaluate the usability and acceptability of the SSRO scaffold across diverse settings, including clinical research, epidemiology, and translational applications. Although the value of the framework will ultimately depend on its uptake and empirical evaluation, we hope the SSRO scaffold provides a practical starting point for organizing resilience research and supports greater conceptual coherence, more consistent measurement, and broader application in medicine.

## Supplementary information

Transparent Peer Review file

Supplementary Methods 1

Description of Additional Supplementary Data

Supplementary Data 1

## References

[1] Krahn, G. L. et al. It’s time to reconsider how we define health: Perspective from disability and chronic condition. *Disabil. Health J.* **14**, 101129 (2021). 34246592 10.1016/j.dhjo.2021.101129

[2] Huber, M. et al. How should we define health? *BMJ* **343**, d4163–d4163 (2011). 21791490 10.1136/bmj.d4163

[3] Scheffer, M. et al. Quantifying resilience of humans and other animals. *Proc. Natl. Acad. Sci. USA*. **115**, 11883–11890 (2018). 30373844 10.1073/pnas.1810630115 PMC6255191

[4] Sturgeon, J. A. & Zautra, A. J. Resilience: A New Paradigm for Adaptation to Chronic Pain. *Curr. Pain. Headache Rep.* **14**, 105–112 (2010). 20425199 10.1007/s11916-010-0095-9 PMC4899321

[5] Stern, Y. et al. A framework for concepts of reserve and resilience in aging. *Neurobiol. Aging* **124**, 100–103 (2023). 36653245 10.1016/j.neurobiolaging.2022.10.015 PMC10424718

[6] Walston, J. et al. A Study of Physical Resilience and Aging (SPRING): Conceptual framework, rationale, and study design. *J. Am. Geriatrics Soc.* **71**, 2393–2405 (2023). 10.1111/jgs.18483 PMC10608799 37386913

[7] Luthar, S. S., Cicchetti, D. & Becker, B. The Construct of Resilience: A Critical Evaluation and Guidelines for Future Work. *Child Dev.* **71**, 543–562 (2000). 10953923 10.1111/1467-8624.00164 PMC1885202

[8] Masten, A. S. Ordinary magic: Resilience processes in development. *Am. Psychologist* **56**, 227–238 (2001). 10.1037//0003-066x.56.3.227 11315249

[9] Bonanno, G. A., Romero, S. A. & Klein, S. I. The Temporal Elements of Psychological Resilience: An Integrative Framework for the Study of Individuals, Families, and Communities. *Psychological Inq.* **26**, 139–169 (2015).

[10] Kalisch, R. et al. The resilience framework as a strategy to combat stress-related disorders. *Nat. Hum. Behav.* **1**, 784–790 (2017). 31024125 10.1038/s41562-017-0200-8

[11] Windle, G. What is resilience? A review and concept analysis. *Rev. Clin. Gerontol.* **21**, 152–169 (2011).

[12] Ungar, M. The social ecology of resilience: Addressing contextual and cultural ambiguity of a nascent construct. *Am. J. Orthopsychiatry* **81**, 1–17 (2011). 21219271 10.1111/j.1939-0025.2010.01067.x

[13] McEwen, B. S. Stress and the Individual: Mechanisms Leading to Disease. *Arch. Intern Med*. **153**, 2093 (1993). 8379800

[14] Romero, L. M., Dickens, M. J. & Cyr, N. E. The reactive scope model — A new model integrating homeostasis, allostasis, and stress. *Hormones Behav.* **55**, 375–389 (2009). 10.1016/j.yhbeh.2008.12.009 19470371

[15] Carpenter, S., Walker, B., Anderies, J. M. & Abel, N. From Metaphor to Measurement: Resilience of What to What? *Ecosystems* **4**, 765–781 (2001).

[16] Heino, M. T. J., Proverbio, D., Marchand, G., Resnicow, K. & Hankonen, N. Attractor landscapes: a unifying conceptual model for understanding behaviour change across scales of observation. *Health Psychol. Rev.* **17**, 655–672 (2023). 36420691 10.1080/17437199.2022.2146598 PMC10261543

[17] Gobbens, R. J. J., Luijkx, K. G., Wijnen-Sponselee, M. T. H. & Schols, J. M. G. A. In Search of an Integral Conceptual Definition of Frailty: Opinions of Experts. *J. Am. Med. Dir. Assoc.* **11**, 338–343 (2010). 20511101 10.1016/j.jamda.2009.09.015

[18] Cesari, M., Gambassi, G., Abellan van Kan, G. & Vellas, B. The frailty phenotype and the frailty index: different instruments for different purposes. *Age Ageing* **43**, 10–12 (2014). 24132852 10.1093/ageing/aft160

[19] Denckla, C. A. et al. Psychological resilience: an update on definitions, a critical appraisal, and research recommendations. *Eur. J. Psychotraumatology* **11**, 1822064 (2020). 10.1080/20008198.2020.1822064 PMC7678676 33244362

[20] Whitson, H. E. et al. Physical Resilience in Older Adults: Systematic Review and Development of an Emerging Construct. *GERONA* **71**, 489–495 (2016). 10.1093/gerona/glv202 PMC5014191 26718984

[21] Resnick, B., Galik, E., Dorsey, S., Scheve, A. & Gutkin, S. Reliability and Validity Testing of the Physical Resilience Measure. * Gerontologist* **51**, 643–652 (2011). 21402647 10.1093/geront/gnr016

[22] Hu, F.-W., Lin, C.-H., Yueh, F.-R., Lo, Y.-T. & Lin, C.-Y. Development and psychometric evaluation of the Physical Resilience Instrument for Older Adults (PRIFOR). *BMC Geriatr.* **22**, 229 (2022). 35313802 10.1186/s12877-022-02918-7 PMC8935854

[23] Ferrucci, L., Levine, M. E., Kuo, P.-L. & Simonsick, E. M. Time and the Metrics of Aging. *Circulation Res.* **123**, 740–744 (2018). 30355074 10.1161/CIRCRESAHA.118.312816 PMC6205734

[24] Ellmers, T. J. et al. Does concern about falling predict future falls in older adults? A systematic review and meta-analysis. *Age Ageing* **54**, afaf089 (2025). 40197783 10.1093/ageing/afaf089 PMC11976718

[25] O’ Brien, H., O’ Leary, N., Scarlett, S., O’ Hare, C. & Kenny, R. A. Hospitalisation and surgery: are there hidden cognitive consequences? Evidence from The Irish Longitudinal study on Ageing (TILDA). *Age Ageing* **47**, 408–415 (2018). 29546387 10.1093/ageing/afy020

[26] Engel, G. L. The Need for a New Medical Model: A Challenge for Biomedicine. *Science* **196**, 129–136 (1977). 847460 10.1126/science.847460

[27] Brown, L., Cohen, B., Costello, R., Brazhnik, O. & Galis, Z. Conceptualizing a resilience research framework at The National Institutes of Health. *Stress Health* **39**, 4–9 (2023). Sep. 37182211 10.1002/smi.3260

[28] S. A. McDorman, E. K. Taylor-Robinette, and R. R. Romeo, Risk and resilience models in child development, in *Advances in Child Development and Behavior*, **67**, Elsevier, 2024, pp. 132–163. 10.1016/bs.acdb.2024.06.005. 10.1016/bs.acdb.2024.06.005 39260902

[29] Cousins, L. A., Kalapurakkel, S., Cohen, L. L. & Simons, L. E. Topical Review: Resilience Resources and Mechanisms in Pediatric Chronic Pain. *J. Pediatr. Psychol.* **40**, 840–845 (2015). 25979085 10.1093/jpepsy/jsv037 PMC4643616

[30] Agostini, L., Onofrio, R., Piccolo, C. & Stefanini, A. A management perspective on resilience in healthcare: a framework and avenues for future research. *BMC Health Serv. Res*. **23**, 774 (2023). 37468875 10.1186/s12913-023-09701-3 PMC10357696

[31] Ferrucci, L., Donega, S., Maier, A. B. & Kroemer, G. Encouraging a move toward precision geromedicine. *Geromedicine*10.70401/Geromedicine.2026.0020 (2025). 10.70401/geromedicine.2026.0020 PMC13246161 42266612

[32] Waugh, C. E. & Koster, E. H. W. A resilience framework for promoting stable remission from depression. *Clin. Psychol. Rev.* **41**, 49–60 (2015). 24930712 10.1016/j.cpr.2014.05.004

[33] Bonanno, G. A. Loss, Trauma, and Human Resilience: Have We Underestimated the Human Capacity to Thrive After Extremely Aversive Events? *Am. Psychologist* **59**, 20–28 (2004). 10.1037/0003-066X.59.1.20 14736317

[34] Fox, C. & Deakin, J. Breaking good? Young people’s mechanisms of resilience, resistance and control. *Br. J. Sociol.* **76**, 316–335 (2025). 39572405 10.1111/1468-4446.13164 PMC11890428

[35] Fernández Castillo, G. et al. The team resilience prescription: Navigating adaptive and maladaptive processes in healthcare teams. *J. Interprof. Care* **39**, 314–320 (2025). 39955780 10.1080/13561820.2025.2460477 PMC11932645

[36] IJntema, R. C., Schaufeli, W. B. & Burger, Y. D. Resilience mechanisms at work: The psychological immunity-psychological elasticity (PI-PE) model of psychological resilience. *Curr. Psychol.* **42**, 4719–4731 (2023). 33994759 10.1007/s12144-021-01813-5 PMC8106546

[37] Cohen, A. A. et al. A complex systems approach to aging biology. *Nat. Aging* **2**, 580–591 (2022). 37117782 10.1038/s43587-022-00252-6 PMC12007111

[38] Koivunen, K. et al. Maintenance of high quality of life as an indicator of resilience during COVID-19 social distancing among community-dwelling older adults in Finland. *Qual. Life Res*. **31**, 713–722 (2022). 34570331 10.1007/s11136-021-03002-0 PMC8475423

[39] Huisman, M., Klokgieters, S. S. & Beekman, A. T. F. Successful ageing, depression and resilience research; a call for a priori approaches to investigations of resilience. *Epidemiol Psychiatr Sci*. **26**, 574–578 (2017). 10.1017/S2045796017000348 PMC6999036 28689499

[40] Cohen, S. & Wills, T. A. Stress, social support, and the buffering hypothesis. *Psychological Bull.* **98**, 310–357 (1985). 3901065

[41] Rector, J. L. et al. Dynamical indicators of resilience from physiological time series in geriatric inpatients: Lessons learned. *Exp. Gerontol.* **149**, 111341 (2021). 33838217 10.1016/j.exger.2021.111341

[42] Conde, C. I. et al. Triggers for freezing of gait in individuals with Parkinson’s disease: a systematic review. *Front. Neurol.* **14**, 1326300 (2023). 38187152 10.3389/fneur.2023.1326300 PMC10771308

