# Supplementary material for: Using the Stressor-System-Resilience Resources-Outcomes (SSRO) scaffold to clarify the core components of resilience research in medicine
Source: Commun Med (Lond). 2026 Aug 15;6:442. doi: 10.1038/s43856-026-01864-4 (PMC13477472; doi:10.1038/s43856-026-01864-4)
Supplement: Supplementary file 1 [file 43856_2026_1864_MOESM1_ESM.pdf]

nature portfolio

Peer Review File

Using the Stressor-System-Resilience Resources-Outcomes (SSRO) scaffold to clarify the core components of resilience research in medicine

Corresponding Author: Dr Deepak Ravi

This file contains all reviewer reports in order by version, followed by all author rebuttals in order by version.

Version 0:

Reviewer comments:

Reviewer #1

(Remarks to the Author)

Thank you for the opportunity to review this manuscript. The authors are to be commended for engaging with an important and interesting area of inquiry, and one that addresses a complex aspect of healthcare.
This paper brings together thinking around the concept of resilience as a whole person approach to understanding how people cope with adversity within a health setting. It positions resilience as a phenomenon and an outcome that can be understood as a whole person experience, similar to frailty. A key development within this essay is a proposed framework to understand the various elements that contribute to resilience. This focus on resilience within health that does not separate out psychological or physical resilience from each other is an important addition to the literature. The focus on stressors in this paper also is a welcome intersection of the stress research with understanding drivers impacting health. The additions of multidimensional spectra or dynamics to both the outcomes and the system aspects of the proposed framework are also important theoretical additions.
That said, in its current form the manuscript would benefit from significant revision prior to being suitable for publication. The comments below are intended to be constructive and to assist the authors in strengthening the clarity, rigor, and overall impact of the work.
The overall validity of this paper could be improved by a more robust exploration and discussion of the controversies around the current definitions of resilience. In particular this paper would benefit from considering definitions from Curtis and Cicchetti whose definition aligns beautifully with this paper: ; “a dynamic process that is influenced by both neural and psychological self-organisations, as well as the transaction between the ecological context and the developing organism” (quoted in Vella SL, Pai NB. A theoretical review of psychological resilience: Defining resilience and resilience research over the decades. Archives of Medicine and Health Sciences. 2019 Jul 1;7(2):233-9.). Positioning the definition of resilience in relationship to key researchers into psychological resilience such as George Bonanno, and in relationship to physiological researchers who use the concept of allostatic load would also strengthen this paper.
We would note that the literature review is relatively limited and does not adequately situate the framework within the existing body of work. A more comprehensive and critical engagement with relevant literature is required to justify the need for the framework and demonstrate how it builds on current literature.
In particular, as this framework builds on the work of the NIH, a robust critique would be required in order to justify modifying that existing published work. We would especially note that conceptualisation of the ‘system’ in the NIH model is more robust and complete - including biological, psychosocial, and spiritual elements. The loss of this detail in the current framework is a significant limitation - especially without clear justification.

Although this topic is very relevant, it is hard to see from this paper how it adds to the current literature beyond the NIH framework. We are also concerned that whilst the manuscript presents a proposed framework that addresses an important and relevant topic, the process by which this framework was developed is not sufficiently described. At present, there is no clear or transparent methodology outlining how the framework was derived, including the data sources, analytical approach, or theoretical underpinnings that informed its development. This limits the reader’s ability to assess the rigor and credibility of the framework.
One final comment is the comparison of this proposed framework to the widely acclaimed and understood PICO framework, it is our opinion that although it communicates vision, this comparison is premature within the scientific piece of writing.

Thank you for the opportunity to review this paper.

Reviewer #2

(Remarks to the Author)
I co-reviewed this manuscript with one of the reviewers who provided the listed reports. This is part of the Communications Medicine initiative to facilitate training in peer review and to provide appropriate recognition for Early Career Researchers who co-review manuscripts.

Reviewer #3

(Remarks to the Author)
Thank you for the invitation to review this paper. It is overall well written.
In my view, the claim the paper makes is overambitious. There have been many attempts to define resilience, in different contexts or overall. While the paper seems to claim it is universally applicable, just two examples are given, both from caring for older adults, and these are very short. Authors could either strive to show its applicability in a wide variety of fields to make the claim that it is "generic". On the other hand, if one wants to establish a simple but universally applicable framework, one would probably need to discuss thoroughly the literature on each of the elements, from different disciplinary perspectives, which I do not see here.
On a side note, I do not think that "resilience characteristics" is a good wording, as one could also think that the elements are characteristics of resilience. What is meant in the text is rather the determinants of factors that make the system more or less resilient.

Version 1:

Reviewer comments:

Reviewer #1

(Remarks to the Author)
Thankyou for the opportunity to read your revised manuscript.
I agree I think it is much strengthened by your responses to both reviewers.

Reviewer #2

(Remarks to the Author)
I co-reviewed this manuscript with one of the reviewers who provided the listed reports. This is part of the Communications Medicine initiative to facilitate training in peer review and to provide appropriate recognition for Early Career Researchers who co-review manuscripts.

Reviewer #3

(Remarks to the Author)
Thank you for inviting me to review the revision of the paper. The authors have responded constructively to the previous round of reviews. The rebuttal is clear and directly addresses the main concerns raised. The addition of Table 1 (mapping 25 resilience traditions onto the four elements), the systematic literature screening description, the change in terminology from “framework” to “scaffold”, the removal of the PICO analogy, and the new section on study design considerations plus Figure 2 (research journey roadmap) represent meaningful improvements. The positioning relative to the NIH resilience framework and other traditions is now clearer and more modest.The manuscript is now a more balanced conceptual perspective piece. However, a few remaining issues should be addressed:

1.Scope and illustrative examples
The two worked examples remain exclusively from geriatric/ageing research (social distancing in community-dwelling older adults and acute illness in hospitalized older adults). While the rebuttal states that these are presented as illustrative rather than evidence of universal applicability, the main text and abstract still frame the SSRO scaffold as relevant across medicine. Table 1 demonstrates breadth across disciplines, but the concrete examples do not. Either expand the examples to include at least one non-geriatric case (e.g., chronic pain, oncology, or acute care in younger adults) or further tone down claims of broad applicability in the abstract, introduction, and conclusion. Currently there is a mismatch between the stated scope and the empirical illustrations provided.

2.Contribution and novelty
The core contribution is a clear organizing scaffold and a useful checklist or roadmap for researchers new to the area. This is valuable for teaching and for improving consistency in how resilience studies are designed and reported. However, the paper remains largely synthetic. The authors should state more explicitly in the introduction and conclusion what new insight or practical advance this scaffold offers beyond existing syntheses (including the NIH framework, which is now appropriately cited). At present the added value is presented somewhat generally (“enhances clarity, comparability, and translation”).

3.Systematic review description
The Supplementary Methods section on the ASReview screening is transparent and appropriate for its purpose (supporting identification of the four recurring element). The description should make clear that this was a targeted, --assisted screening exercise stopped at ~10% coverage plus a stopping rule, not a full systematic review of the resilience literature. This distinction is important to avoid overstatement.

4.The conclusion still contains some repetitive phrasing about the benefits of the scaffold (clarity, comparability, translation). Tighten this section; it currently feels slightly promotional.

5.Figure 2 (research journey roadmap) is a helpful addition . Consider whether it can be referenced earlier in the text when the need for guidance for new researchers is first mentioned.

Version 2:

Reviewer comments:

Reviewer #3

(Remarks to the Author)
Thank you for addressing all points.
My last comment is that the Conclusions should be more modest. Phrases like "Establishing SSRO as a common scaffold … will lay the foundation for a more coherent science…" is too bold a statement IMHO

Open Access This Peer Review File is licensed under a Creative Commons Attribution 4.0 International License, which permits use, sharing, adaptation, distribution and reproduction in any medium or format, as long as you give appropriate credit to the original author(s) and the source, provide a link to the Creative Commons license, and indicate if changes were made.
In cases where reviewers are anonymous, credit should be given to 'Anonymous Referee' and the source.
The images or other third party material in this Peer Review File are included in the article's Creative Commons license, unless indicated otherwise in a credit line to the material. If material is not included in the article's Creative Commons license and your intended use is not permitted by statutory regulation or exceeds the permitted use, you will need to obtain permission directly from the copyright holder.
To view a copy of this license, visit https://creativecommons.org/licenses/by/4.0/

Response to Reviewers

The Authors would like to thank the Reviewers for their feedback to our manuscript. A brief point-by-point response to Reviewers' comments is presented below. To ensure clarity throughout, the Reviewers' comments are presented in blue, with our responses in an indented format and a black font. All changes made to the manuscript are presented under quotes in black font and highlighted in yellow and the **corresponding page and line numbers (in the clean version)** of the revised manuscript are denoted in red and bold.

Reviewer #1 (Remarks to the Author):

Thank you for the opportunity to review this manuscript. The authors are to be commended for engaging with an important and interesting area of inquiry, and one that addresses a complex aspect of healthcare.

This paper brings together thinking around the concept of resilience as a whole person approach to understanding how people cope with adversity within a health setting. It positions resilience as a phenomenon and an outcome that can be understood as a whole person experience, similar to frailty. A key development within this essay is a proposed framework to understand the various elements that contribute to resilience. This focus on resilience within health that does not separate out psychological or physical resilience from each other is an important addition to the literature. The focus on stressors in this paper also is a welcome intersection of the stress research with understanding drivers impacting health. The additions of multidimensional spectra or dynamics to both the outcomes and the system aspects of the proposed framework are also important theoretical additions.

> We sincerely thank the reviewer for their thoughtful and generous assessment of the manuscript. We are particularly encouraged that the reviewer found the whole-person framing of resilience and the structural-to-dynamic spectrum of resilience-relevant resources to be meaningful contributions. We have worked to strengthen the manuscript further in response to the specific concerns raised below.

That said, in its current form the manuscript would benefit from significant revision prior to being suitable for publication. The comments below are intended to be constructive and to assist the authors in strengthening the clarity, rigor, and overall impact of the work.

The overall validity of this paper could be improved by a more robust exploration and discussion of the controversies around the current definitions of resilience. In particular this paper would benefit from considering definitions from Curtis and Cicchetti whose definition aligns beautifully with this paper: ; “a dynamic process that is influenced by both neural and psychological self- organisations, as well as the transaction between the ecological context and the developing organism” (quoted in Vella SL, Pai NB. A theoretical review of psychological resilience: Defining resilience and resilience research over the decades. Archives of Medicine and Health Sciences. 2019 Jul 1;7(2):233-9.). Positioning the definition of resilience in relationship to key researchers into psychological resilience such as George Bonanno, and in relationship to physiological researchers who use the concept of allostatic load would also strengthen this paper. We would note that the literature review is relatively limited and does not adequately situate the framework within the existing body of work. A more comprehensive and critical engagement with relevant literature is required to justify the need for the framework and demonstrate how it builds on current literature.

We agree with this comment and thank the reviewer for the specific suggestions. We have substantially expanded our engagement with the broader resilience literature. The revised manuscript now includes explicit discussion of developmental psychology perspectives associated with Cicchetti and colleagues and Masten, psychological resilience traditions associated with Bonanno and colleagues and Kalisch et al., and physiological adaptation traditions including allostasis and allostatic load (McEwen and Stellar; Romero et al.). These traditions are discussed in the section 'Resilience Across Disciplines: Why Greater Conceptual Clarity Is Needed' and are systematically mapped onto the four SSRO elements in the newly added Table 1.

We also clarify that the SSRO scaffold is not intended as a new definition or framework of resilience, but rather as a simple organizing structure that makes recurring elements across existing traditions more visible and explicit. To avoid any confusion with existing frameworks, we refer to the SSRO consistently as a scaffold throughout the revised manuscript.

The following passages from the revised manuscript reflect these additions:

(Page 2 Line 71) “Developmental and psychological perspectives have emphasized resilience as positive adaptation in the context of adversity, often highlighting dynamic processes that unfold over time [7]–[10]. Social-ecological perspectives have further stressed that resilience depends not only on the individual but also on access to social, environmental, and cultural resources [11], [12]. Physiological traditions, including allostasis and allostatic load, have framed adaptation in terms of dynamic regulation and the cumulative costs of repeated stress responses [13], [14]. Ecological perspectives, in turn, have emphasized resilience as the amount of disturbance a complex system can tolerate before shifting to another stable state. This tradition also makes clear that resilience is not inherently positive, since systems may remain resilient in both desirable and undesirable states [15], [16].”

Table 1 **(Page 4 to 9)** was further added to provide a systematic mapping of 25 representative resilience traditions across disciplines onto the four SSRO elements,

illustrating that despite differences in terminology and emphasis, stressor, system, resilience-relevant resources and processes, and outcomes recur across the field. The synthesis presented in Table 1 was informed by a systematic literature review using machine learning-based active learning (see Supplementary Methods).

(Page 21 Line 557) **"Supplementary Methods 1: Systematic Literature Review in Support of Table 1**

To support the identification of four recurring elements across resilience traditions, we conducted a systematic literature review using machine learning-based active learning. We searched PubMed using the following strategy: "resilience framework" [tiab: ~10] OR "resilience model" [tiab: ~10] OR "resilienc* framework*"[tiab] OR "resilienc* model*"[tiab]. This yielded 5,654 unique records. We used ASReview LAB to screen records based on title and abstract. ASReview LAB applies active learning, in which a machine learning model is continuously retrained based on the labeller's selections, progressively reranking remaining records to improve screening efficiency. Screening was stopped when at least 10% of all records had been reviewed and a stopping threshold of more than 100 consecutively unselected records had been reached. The findings of the review supported the four recurring elements: stressor, system, resilience-relevant resources and processes, and outcomes across resilience traditions in multiple disciplines, and directly informed the synthesis presented in Table 1."

In particular, as this framework builds on the work of the NIH, a robust critique would be required in order to justify modifying that existing published work. We would especially note that conceptualisation of the 'system' in the NIH model is more robust and complete - including biological, psychosocial, and spiritual elements. The loss of this detail in the current framework is a significant limitation - especially without clear justification. Although this topic is very relevant, it is hard to see from this paper how it adds to the current literature beyond the NIH framework.

We thank the reviewer for raising this important point and apologize for the lack of clarity in the original manuscript. Our intention was never to modify or replace the NIH framework, nor any other existing resilience framework. Rather, the SSRO scaffold is intended to complement existing frameworks by offering a simpler and more accessible organizing structure in a field where the wealth of existing models can itself be overwhelming.

We fully agree that the NIH framework's conceptualization of the system, including biological, psychosocial, and spiritual elements, is comprehensive and important. In the revised manuscript, we make explicit that the system element of the SSRO may encompass biological, psychological, cognitive, social, and, where relevant, spiritual dimensions, as well as their interconnections. This aligns fully with, and does not diminish, the breadth of the NIH framework. The impression to the contrary in the original manuscript was unintended and arose partly because our worked examples were drawn from clinical and geriatric contexts, where organ systems and physiological processes are the most immediately recognizable entry point for the target audience.

The NIH framework is now explicitly cited as a key antecedent in the revised manuscript, and it is included in Table 1 alongside 24 other resilience traditions. The following revised passage makes our relationship to the NIH framework explicit:

(Page 10 Line 152) "Building on prior work across resilience research, including the trans-NIH Resilience Working Group framework [27] and other lines of work, such as those on pain resilience [4], we argue that resilience research in medicine benefits from a simple cross-cutting structure. At a minimum, this structure should specify (1) the stressor or challenge, (2) the system under consideration (often the individual), (3) the resilience-relevant resources and processes within or around the system that are hypothesized to shape its response to the stressor, and (4) the outcomes of interest (Figure 1). We refer to this organizing scaffold as the SSRO - Stressor, System, Resilience-relevant resources/processes, and Outcomes. These four elements form the essential building blocks of resilience research. Each may be physical, psychological, cognitive, or social in nature, and they often interact across domains. SSRO is designed as a simple and memorable organizing scaffold and educational tool to promote clarity, comparability, and rigor in resilience research"

(Page 11 Line 199) "When defining the system, it is therefore important to consider not only the individual and their internal resources, but also the broader ecological, social, and temporal context in which they are embedded. Omitting this context risks reducing resilience to a property of the person alone, which both misrepresents the underlying process and limits the scope for intervention."

We are also concerned that whilst the manuscript presents a proposed framework that addresses an important and relevant topic, the process by which this framework was developed is not sufficiently described. At present, there is no clear or transparent methodology outlining how the framework was derived, including the data sources, analytical approach, or theoretical underpinnings that informed its development. This limits the reader's ability to assess the rigor and credibility of the framework.

We agree that the developmental process of the SSRO scaffold should have been described more transparently, and we have addressed this in the revised manuscript. We clarify that this article is a conceptual perspective rather than a framework-development study using a structured methodology, and that the SSRO scaffold emerged through iterative interdisciplinary discussion among co-authors with complementary expertise in geriatrics, rehabilitation, aging, and resilience research, informed by synthesis of resilience research from fields within and adjacent to medicine. We also added a systematic literature review (described in the Supplementary Methods 1) that was conducted to support the identification of four recurring elements across resilience traditions and directly informed the synthesis presented in Table 1. The following passage from the Introduction now describes the development process:

(Page 2 Line 58) "This perspective was developed through iterative interdisciplinary discussions among the co-authors, informed by their complementary expertise in

geriatrics, rehabilitation, aging, and resilience research. It was further informed by synthesis of resilience research from fields within and adjacent to medicine, including psychology, ecology, and engineering, with a particular focus on insights applicable to somatic health."

Additionally, the Supplementary Methods section describes the systematic literature review that was conducted to support the identification of the four recurring elements:

(Page 21 Line 557) "Supplementary Methods 1: Systematic Literature Review in Support of Table 1

To support the identification of four recurring elements across resilience traditions, we conducted a systematic literature review using machine learning-based active learning. We searched PubMed using the following strategy: "resilience framework" [tiab: ~10] OR "resilience model" [tiab: ~10] OR "resilienc* framework*"[tiab] OR "resilienc* model*"[tiab]. This yielded 5,654 unique records. We used ASReview LAB to screen records based on title and abstract. ASReview LAB applies active learning, in which a machine learning model is continuously retrained based on the labeller's selections, progressively reranking remaining records to improve screening efficiency. Screening was stopped when at least 10% of all records had been reviewed and a stopping threshold of more than 100 consecutively unselected records had been reached. The findings of the review supported the four recurring elements: stressor, system, resilience-relevant resources and processes, and outcomes across resilience traditions in multiple disciplines, and directly informed the synthesis presented in Table 1."

One final comment is the comparison of this proposed framework to the widely acclaimed and understood PICO framework, it is our opinion that although it communicates vision, this comparison is premature within the scientific piece of writing.

We agree with the reviewer and have removed the comparison to the PICO framework from the revised manuscript. We now describe SSRO more modestly as a conceptual scaffold intended to improve clarity, comparability, and rigor in resilience research, without drawing analogies to established clinical tools that may set expectations we cannot yet substantiate.

Thank you for the opportunity to review this paper.

Reviewer #3 (Remarks to the Author):

Thank you for the invitation to review this paper. It is overall well written.

We thank the reviewer for their careful reading and constructive comments.

In my view, the claim the paper makes is overambitious. There have been many attempts to define resilience, in different contexts or overall.

We agree that the original manuscript overstated the scope of the SSRO. Our intention was never to offer a new universal definition of resilience, nor to compete with or replace existing resilience frameworks. In the revised manuscript, we have consistently positioned the SSRO as a practical organizing scaffold that complements existing approaches making it easier to structure, compare, and communicate resilience research rather than as a new definition or framework. We have softened the language accordingly throughout the manuscript, and we believe the addition of Table 1, which explicitly maps 25 established resilience traditions onto the four SSRO elements, demonstrates that these elements emerge from the existing literature rather than being imposed upon it.

While the paper seems to claim it is universally applicable, just two examples are given, both from caring for older adults, and these are very short. Authors could either strive to show its applicability in a wide variety of fields to make the claim that it is "generic".

We agree that two ageing-related examples are insufficient to support a strong claim of universal applicability. In the revised manuscript, we have addressed this in two ways.

First, we explicitly soften the claim, presenting the two worked examples as illustrative case studies rather than as evidence of universal applicability, and acknowledging that the authors' expertise is primarily rooted in ageing, falls, rehabilitation, and clinical care.

(Page 2 Line 58) "This perspective was developed through iterative interdisciplinary discussions among the co-authors, informed by their complementary expertise in geriatrics, rehabilitation, aging, and resilience research. It was further informed by synthesis of resilience research from fields within and adjacent to medicine, including psychology, ecology, and engineering, with a particular focus on insights applicable to somatic health."

Second, and more substantially, we have added Table 1, which maps 25 representative resilience traditions from developmental science, psychology, ecology, engineering, public health, pain research, gerontology, and clinical medicine onto the four SSRO elements. This table demonstrates that the elements recur across a wide range of disciplines, without relying solely on the ageing examples. The table caption explicitly notes:

(Page 4 Line 125) "Influential resilience traditions across disciplines differ in terminology and emphasis yet commonly converge on four recurring elements: a stressor, the system under consideration, resilience-relevant resources and processes,

and outcomes. The table is not intended to be exhaustive but to illustrate this convergence across a representative selection of traditions."

On the other hand, if one wants to establish a simple but universally applicable framework, one would probably need to discuss thoroughly the literature on each of the elements, from different disciplinary perspectives, which I do not see here.

We appreciate this observation. Given that our goal is to offer a simple and accessible organizing scaffold rather than a comprehensive review, we have opted to engage with each element substantively rather than exhaustively. The revised manuscript now devotes dedicated subsections to each of the four SSRO elements: Stressors, System, Resilience-relevant resources and processes, and Outcomes, with cross-disciplinary discussion drawing on developmental, psychological, ecological, physiological, and clinical perspectives. We believe this level of engagement is appropriate for a conceptual perspective and is substantially more than was present in the original manuscript. A fully exhaustive literature review of each element across all disciplines would be beyond the scope of this format and is better suited to a systematic review.

The revised discussion of the elements now includes:

Stressors

(Page 10 Line 171) "The terminology used to describe the stressor varies considerably across resilience traditions, reflecting differences in disciplinary context. In human resilience research, the terms adversity, challenge, and stressor are most common, while the ecology literature favors perturbation, and engineering uses shock or crisis. Some traditions also frame the stressor as a risk [36], [41]. This terminological variation has substantive implications. Framing the stressor as a risk connects resilience research to risk management, where proactive preparedness aims to reduce the likelihood or impact of an adverse event before it occurs. Resilience management, in contrast, focuses on reactive preparedness - minimizing the impact of a stressor once it materializes. These two approaches are complementary rather than competing [37]. Additionally, the word stressor implies a temporal dimension, a discrete exposure with a beginning and an end, whereas the word risk carries no such implication. This distinction underscores that stressors require adequate characterization not only in terms of type and magnitude, but also duration. Finally, framing the stressor as a risk ties it to questions of causality. While the literature on causal evaluation of risk factors is well developed, causal study of resilience factors remains sparse, with most resilience research oriented toward prediction rather than causal inference. Yet for the purposes of intervention, understanding the causal role of resilience factors and mechanisms is essential."

System

(Page 11 Line 195) "There is general consensus in the resilience literature that resilience is not a fixed individual trait, but rather a process shaped by the ongoing interaction between the individual and their environment across space and time. Nevertheless, research practice does not always reflect this consensus, and resilience

is sometimes studied through a set of cross-sectionally collected individual characteristics. When defining the system, it is therefore important to consider not only the individual and their internal resources, but also the broader ecological, social, and temporal context in which they are embedded. Omitting this context risks reducing resilience to a property of the person alone, which both misrepresents the underlying process and limits the scope for intervention."

Outcomes

(Page 13 Line 254) "An important consideration in operationalizing outcomes concerns the valence attributed to resilience. In everyday language, and often in psychological and stress resilience research, resilience is understood as inherently positive, an outcome is only considered resilient if it involves growth or improvement beyond pre-adversity functioning. However, this positive valence is not a universal feature of resilience. In ecological conceptualizations, both desired and undesired stable states can be resilient, since resilience refers to the persistence of a system state, not its desirability [11], [15]. This has meaningful clinical implications. There is no such thing as a universally resilient person; resilience is not a static, unitary property exclusive to those without deficits or pathology [7], [43], [44]. A frail person can exhibit resilience, and that process can be supported. Even responses that appear maladaptive or normatively deviant in one domain may serve to maintain integrity in another domain or in the system as a whole [45], [46]. These considerations further underscore the importance of explicit and careful operationalization of outcomes in resilience research.

A related concept that merits attention is the distinction between psychological immunity and psychological elasticity, which connects to the broader phenomenon of steeling versus sensitization [39]. The same adversity may either strengthen the system's response repertoire, steeling, or weaken it through sensitization. This means that exposure to a stressor does not have a fixed or predictable effect on future resilience; the direction depends on the nature of the stressor, the system, and the context. Recognizing this bidirectionality reinforces the importance of longitudinal outcome assessment and careful specification of the timeframe over which resilience is evaluated."

On a side note, I do not think that "resilience characteristics" is a good wording, as one could also think that the elements are characteristics of resilience. What is meant in the text is rather the determinants of factors that make the system more or less resilient.

We thank the reviewer for this helpful observation. We agree that 'resilience characteristics' is ambiguous and could be misread as referring to properties of the resilience response itself rather than to features of the system that shape how it responds to a stressor. In the revised manuscript, we have replaced this term with 'resilience-relevant resources and processes' throughout. This phrasing clarifies that we are referring to system features, determinants, or processes that may make a system more or less resilient in response to a given stressor, not to resilience outcomes, and aligns with our distinction between more structural and more dynamic indicators. The following passage from the revised manuscript illustrates the updated terminology:

(Page 11 Line 205) "In principle, these refer to features of the system of interest that are hypothesized to shape how the system responds to a stressor. However, resources external to the system, such as social welfare support or community infrastructure, may also enhance its resilient response. Within the SSRO scaffold, such external factors can be considered contextual resources that interact with, rather than belong to, the system itself, depending on the exact delineation of the system of interest. We also propose distinguishing between resilience-relevant resources and processes along a spectrum from more structural or static indicators to more dynamic indicators."

We hope these revisions adequately address the concerns raised by both reviewers. We believe the manuscript is substantially strengthened by the addition of Table 1, the more transparent description of the development process, the expanded engagement with the broader resilience literature, the consistent use of 'scaffold' throughout, and the addition of the research journey roadmap for scholars new to the field. We remain grateful for the constructive feedback and look forward to the reviewers' assessment.

Response to Reviewers

The Authors would like to thank the Reviewers for their feedback to our manuscript. A brief point-by-point response to Reviewers' comments is presented below. To ensure clarity throughout, the Reviewers' comments are presented in blue, with our responses in an indented format and a black font. All changes made to the manuscript are presented under quotes in black font and highlighted in yellow.

Response to Reviewers

Reviewers' comments:

Reviewer #1 (Remarks to the Author):

Thank you for the opportunity to read your revised manuscript. I agree I think it is much strengthened by your responses to both reviewers.

> We thank Reviewer 1 for this positive assessment of the revised manuscript.

Reviewer #2 (Remarks to the Author):

I co-reviewed this manuscript with one of the reviewers who provided the listed reports. This is part of the Communications Medicine initiative to facilitate training in peer review and to provide appropriate recognition for Early Career Researchers who co-review manuscripts.

> We thank Reviewer 2 for their contribution to the review

process. Reviewer #3 (Remarks to the Author):

Thank you for inviting me to review the revision of the paper. The authors have responded constructively to the previous round of reviews. The rebuttal is clear and directly addresses the main concerns raised. The addition of Table 1 (mapping 25 resilience traditions onto the four elements), the systematic literature screening description, the change in terminology from "framework" to "scaffold", the removal of the PICO analogy, and the new section on study design considerations plus Figure 2 (research journey roadmap) represent meaningful improvements. The positioning relative to the NIH resilience framework and other traditions is now clearer and more modest. The manuscript is now a more balanced conceptual perspective piece.

> We thank the reviewer for this positive assessment of our revision and for recognizing the improvements made in response to the previous round of review.

However, a few remaining issues should be addressed:

1.Scope and illustrative examples.

The two worked examples remain exclusively from geriatric/ageing research (social distancing in community-dwelling older adults and acute illness in hospitalized older adults). While the rebuttal states that these are presented as illustrative rather than evidence of universal applicability, the main text and abstract still frame the SSRO scaffold as relevant across

medicine. Table 1 demonstrates breadth across disciplines, but the concrete examples do not. Either expand the examples to include at least one non-geriatric case (e.g., chronic pain,

oncology, or acute care in younger adults) or further tone down claims of broad applicability in the abstract, introduction, and conclusion. Currently there is a mismatch between the stated scope and the empirical illustrations provided.

We thank the reviewer for this important observation. We have added a third worked example illustrating freezing of gait (FOG) in Parkinson's disease. This example broadens the illustrative scope of the manuscript beyond aging-framed contexts in two ways: first, Parkinson's disease and FOG are not intrinsically aging-specific (onset can occur from mid-adulthood), and the example is framed around the neurological mechanism rather than age; second, it demonstrates that the scaffold accommodates stressors that are brief, situational, and recurring (environmental and task-related triggers such as turning, doorways, and dual-tasking), in contrast to the more prolonged stressors in Examples 1 and 2 (social distancing; acute illness). This shows the scaffold's flexibility across different stressor timescales, not only across disciplines. The new example reads:

"Example 3, Resilience to Freezing of Gait in Parkinson's Disease

Freezing of gait (FOG) is a common and disabling symptom of Parkinson's disease, characterized by a sudden, brief inability to generate effective stepping despite the intention to walk. Unlike the stressors in Examples 1 and 2, which are prolonged and largely externally imposed, FOG-triggering stressors are momentary and recur repeatedly within a single day: turning, walking through doorways, dual-tasking, and approaching a destination are all well-established provocateurs (Figure 5) [41].

The system under consideration is the individual's locomotor control network, encompassing cortico-basal ganglia-brainstem circuits for gait initiation and task-switching, which in Parkinson's disease operate under dopaminergic depletion and reduced automaticity, placing greater demand on attentional and executive resources during gait.

Structure indicators relevant to FOG resilience include disease stage, FOG phenotype and history, dopaminergic medication state, executive function, and fear of falling, all of which shape a person's underlying vulnerability to freezing. Dynamic indicators become observable only when the system is challenged by a trigger: gait adaptability during turning or dual-tasking, pre-freeze changes in step length and cadence variability, and the effectiveness of compensatory cueing strategies, such as visual floor markers or auditory rhythmic cues, in restoring stepping. These dynamic indicators most directly capture resilience, since two people with similar disease severity can respond very differently to the same trigger: one recovers stepping within a stride using a cueing strategy, while another enters a prolonged freeze culminating in a near-fall or fall.

Outcomes can therefore be defined at the level of a single episode (resolution versus persistence of the freeze, and whether a fall results) or aggregated over time (FOG frequency and severity, and downstream effects on mobility-related quality of life). This example illustrates how the SSRO scaffold accommodates stressors that are brief and

repeated, extending the scaffold's applicability beyond the longer-timescale stressors featured in Examples 1 and 2.”

Corresponding Figure 5:

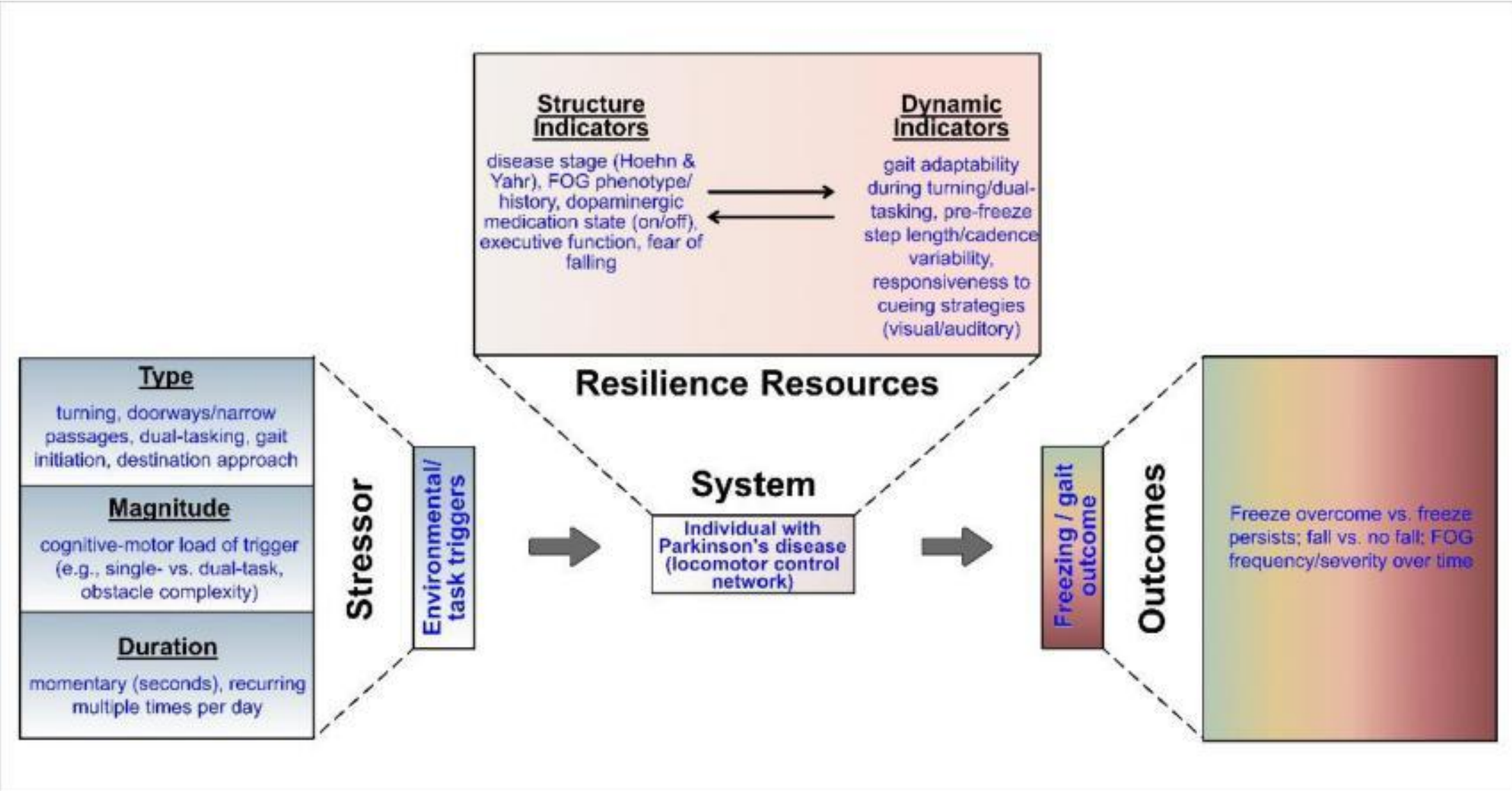

2.Contribution and novelty

The core contribution is a clear organizing scaffold and a useful checklist or roadmap for researchers new to the area. This is valuable for teaching and for improving consistency in how resilience studies are designed and reported. However, the paper remains largely synthetic. The authors should state more explicitly in the introduction and conclusion what new insight or practical advance this scaffold offers beyond existing syntheses (including the NIH framework, which is now appropriately cited). At present the added value is presented somewhat generally (“enhances clarity, comparability, and translation”).

We thank the reviewer for pushing us to be more explicit. We have added a passage to the Introduction, directly adjacent to our citation of the trans-NIH Resilience Working Group framework, stating what SSRO offers beyond existing domain-specific syntheses: its deliberate minimalism, which allows it to function as a common reference structure across the traditions in Supplementary Table 1 rather than compete with them, and its explicit structure/dynamic indicator distinction, which translates into a concrete measurement decision rarely made explicit elsewhere. The added text reads:

"Unlike many domain-specific frameworks summarized in Supplementary Table 1, which are pitched within a particular field and rich in that field's own constructs, the SSRO scaffold is deliberately minimal: it specifies only the four elements that must be defined for resilience to be studied at all, regardless of discipline. This minimalism allows the scaffold to function as a common reference structure that the traditions summarized in Supplementary Table 1 can be mapped onto, making it possible to translate between traditions rather than requiring researchers to adopt one over another."

Please see response to Comment 4 on changes made in the Conclusion.

3.Systematic review description

The Supplementary Methods section on the ASReview screening is transparent and appropriate for its purpose (supporting identification of the four recurring element). The description should make clear that this was a targeted, machine-learning-assisted screening exercise stopped at ~10% coverage plus a stopping rule, not a full systematic review of the resilience literature. This distinction is important to avoid overstatement.

We agree and have revised the text as follows:

“To support the identification of four recurring elements across resilience traditions, we conducted a machine-learning-assisted screening of the literature. We searched PubMed using the following strategy: "resilience framework" [tiab: ~10] OR "resilience model" [tiab: ~10] OR "resilienc* framework*"[tiab] OR "resilienc* model*"[tiab]. This yielded 5,654 unique records. We used ASReview LAB to screen records based on title and abstract, applying active learning in which a machine learning model is continuously retrained based on the labeller's selections, progressively reranking remaining records to improve screening efficiency. This was a targeted screening exercise conducted to support identification of the four recurring elements presented in Table 1, not a full systematic review of the resilience literature. Screening was stopped when at least 10% of all records had been reviewed and a stopping threshold of more than 100 consecutively unselected records had been reached.”

4.The conclusion still contains some repetitive phrasing about the benefits of the scaffold (clarity, comparability, translation). Tighten this section; it currently feels slightly promotional.

We agree and have removed the followed sentences from the Conclusion in its entirety:

“Framing resilience in this way provides a foundation for more coherent research, more reliable measurement, and ultimately, more meaningful application in medicine.”

“The SSRO scaffold provides a simple but flexible structure that can bring several benefits. It enhances clarity, by ensuring that resilience studies specify what challenge is being faced, who or what constitutes the system, and what outcomes are relevant. It improves comparability, by creating a common language across medical studies and contexts. And it supports translation, by making it easier to apply resilience concepts consistently in both research and clinical care.”

Revised conclusion:

“Resilience offers a promising lens for understanding how individuals navigate health challenges across the course of life. To translate this promise into practice, we need simple, clear tools that help to structure resilience research in more consistent ways. At a minimum, studying resilience requires four elements: a defined stressor, a specified system, the resilience-relevant resources and processes that may shape how the system responds, and the outcomes that follow. We refer to this organizing framework as the SSRO scaffold. These components must be considered across physical, psychological, cognitive, and social domains, and in relation to the context in

which adaptation occurs. Using the SSRO scaffold as a starting point, researchers can detail their design using the resilience tradition that best fits their use case. For those new to the field, we additionally offer a step-by-step research journey roadmap to guide key methodological decisions, from conceptual framing to interpretation.

In the future, advancing the field will require consensus among experts to establish unequivocal operational definitions for each of the four SSRO elements. Such agreement may not yet be necessary for conceptual progress but will be essential for developing standardized measures, enabling data synthesis, and ensuring comparability across studies. Future work should also evaluate the usability and acceptability of the SSRO scaffold across diverse settings, including clinical research, epidemiology, and translational applications. Establishing SSRO as a common scaffold for resilience research will lay the foundation for a more coherent science, more reliable measurement, and ultimately, more meaningful application in medicine."

5.Figure 2 (research journey roadmap) is a helpful addition . Consider whether it can be referenced earlier in the text when the need for guidance for new researchers is first mentioned.

We agree and have moved the first reference to Figure 2 earlier, to the point where the SSRO scaffold and its four elements are first fully defined, rather than only at the end of the Study Design Considerations section. The added sentence reads: "To help researchers new to the field translate these elements into practice, we provide a step-by-step research journey roadmap (Figure 2)." We did not move the reference earlier than this point, as Figure 2 depends on the SSRO elements already having been introduced to be meaningful to the reader.

Response to Reviewers

The Authors would like to thank the Reviewers for their feedback to our manuscript. A brief point-by-point response to Reviewers' comments is presented below. To ensure clarity throughout, the Reviewers' comments are presented in blue, with our responses in an indented format and a black font. All changes made to the manuscript are presented under quotes in black font and highlighted in yellow.

Response to Reviewers

Reviewers' comments:

Reviewer #3 (Remarks to the Author):

Thank you for addressing all points. My last comment is that the Conclusions should be more modest. Phrases like "Establishing SSRO as a common scaffold ... will lay the foundation for a more coherent science..." is too bold a statement IMHO

Thank you for this helpful suggestion. We have revised the conclusion to adopt a more cautious tone and moderated the claims regarding the potential impact of the SSRO scaffold. Please see the revised conclusion below:

"Resilience offers a promising lens for understanding how individuals navigate health challenges across the course of life. To translate this promise into practice, resilience research would benefit from simple, clear tools that support more consistent study design and reporting. We propose that resilience research can be organized around four core elements: a defined stressor, a specified system, the resilience-relevant resources and processes that may shape how the system responds, and the outcomes that follow. We refer to this organizing scaffold as SSRO. These components must be considered across physical, psychological, cognitive, and social domains, and in relation to the context in which adaptation occurs. Using the SSRO scaffold as a starting point, researchers can detail their study design according to the resilience tradition that best fits their use case. For those new to the field, we additionally offer a step-by-step research journey roadmap to guide key methodological decisions, from conceptual framing to interpretation.

In the future, advancing the field will ultimately require consensus among experts to establish unequivocal operational definitions for each of the four SSRO elements. Such agreement may not yet be necessary for conceptual progress but will likely be important for developing standardized measures, enabling data synthesis, and improving comparability across studies. Future work should also evaluate the usability and acceptability of the SSRO scaffold across diverse settings, including clinical research, epidemiology, and translational applications. Although the value of the framework will ultimately depend on its uptake and empirical evaluation, we hope the SSRO scaffold provides a practical starting point for organizing resilience research and supports greater conceptual coherence, more consistent measurement, and broader application in medicine."
