# Supplementary material for: Using the Stressor-System-Resilience Resources-Outcomes (SSRO) scaffold to clarify the core components of resilience research in medicine
Source: Commun Med (Lond). 2026 Aug 15;6:442. doi: 10.1038/s43856-026-01864-4 (PMC13477472; doi:10.1038/s43856-026-01864-4)
Supplement: Supplementary file 2 [file 43856_2026_1864_MOESM2_ESM.pdf]

Supplementary Methods 1: Literature Screening in Support of Supplementary Data 1

To support the identification of four recurring elements across resilience traditions, we conducted a machine-learning-assisted screening of the literature. We searched PubMed using the following strategy: "resilience framework" [tiab: ~10] OR "resilience model" [tiab: ~10] OR "resilienc* framework*"[tiab] OR "resilienc* model*"[tiab]. This yielded 5,654 unique records. We used ASReview LAB to screen records based on title and abstract, applying active learning in which a machine learning model is continuously retrained based on the labeller's selections, progressively reranking remaining records to improve screening efficiency. This was a targeted screening exercise conducted to support identification of the four recurring elements presented in Supplementary Data 1, not a full systematic review of the resilience literature. Screening was stopped when at least 10% of all records had been reviewed and a stopping threshold of more than 100 consecutively unselected records had been reached.
