# Supplementary material for: Using the Stressor-System-Resilience Resources-Outcomes (SSRO) scaffold to clarify the core components of resilience research in medicine
Source: Commun Med (Lond). 2026 Aug 15;6:442. doi: 10.1038/s43856-026-01864-4 (PMC13477472; doi:10.1038/s43856-026-01864-4)
Supplement: Supplementary file 3 [file 43856_2026_1864_MOESM3_ESM.docx]

Description of additional supplementary file

File name: Supplementary Data 1

Description: Representative resilience traditions across disciplines and their mapping onto the SSRO scaffold. This supplementary dataset summarizes representative resilience traditions across disciplines and illustrates their convergence around the four recurring SSRO elements (stressor, system, resilience-relevant resources and processes, and outcomes). The synthesis was informed by a machine learning-assisted literature screening described in Supplementary Methods 1.
